# The relationship between protein-energy wasting and cognitive impairment in patients receiving maintenance hemodialysis

**DOI:** 10.3389/fneur.2025.1619083

**Published:** 2025-07-31

**Authors:** Jun Liu, Jingfang Wan, Kehong Chen, Yani He, Weiwei Zhang, Jiyuan Luo, Dan Li

**Affiliations:** Department of Nephrology, Daping hospital, Army Medical Center of PLA, Changjiang Road, Daping, Yuzhong District, Chongqing, China

**Keywords:** cognitive impairment, protein-energy wasting, maintenance hemodialysis, relationship, patients

## Abstract

**Objectives:**

To investigate the relationship between protein-energy wasting (PEW) and cognitive impairment (CI) in patients undergoing maintenance hemodialysis (MHD).

**Methods:**

A total of 185 patients undergoing MHD between June 2020 and April 2022 were initially recruited. Among the initially recruited patients, 25 patients were excluded, and 160 patients were finally involved in this study. The Montreal Cognitive Assessment-Basic (MoCA-B) was utilized to assess patients’ cognitive functions. Patients were categorized into two groups based on the presence or absence of PEW as per the diagnostic criteria. The severity of PEW was evaluated using the Scored Patient-Generated Subjective Global Assessment (PG-SGA) and Malnutrition Inflammation Score (MIS). Baseline data, PEW-related indicators, and cognitive function scores were compared between the CI and non-CI groups. Logistic regression analysis was employed to identify the factors influencing CI in patients undergoing MHD.

**Results:**

There were significant differences between the two groups in terms of age, educational level, Kt/V, PEW detection rate, PEW-related indicators [albumin (ALB)], lean body mass, arm muscle circumference (AMC), normalized dietary protein intake (nDPI), PG-SGA score, and MIS score (*p* < 0.05). The MoCA-B scores of MHD patients with PEW were significantly lower than those of patients without PEW (*p* < 0.05). The two groups exhibited significant differences in executive function, orientation, abstraction, delayed recall, and attention scores (*p* < 0.05). Multivariate logistic regression analysis indicated that age, PEW severity, Kt/V, and ALB were significant influential factors of CI in patients undergoing MHD (*p* < 0.05).

**Conclusion:**

PEW was closely associated with CI in patients undergoing MHD. Those combined with PEW were more likely to develop CI, and to suffer from more severe CI. Active precautionary measures should be taken against PEW to delay the progression of CI in patients undergoing MHD.

## 1 Introduction

Maintenance hemodialysis (MHD) is the primary treatment for patients with end-stage renal disease (ESRD). While hemodialysis significantly extends MHD patients’ lifespan, there has been a significant increase in the incidence of several co-morbidities (1). Cognitive impairment (CI), frequently recognized as a co-morbidity in patients undergoing MHD, encompasses a range of disorders that impact executive functions, as well as memory, orientation, and language abilities. These impairments can arise from various factors. Patients with CI undergoing MHD mainly experience a decrease in their overall quality of life. Additionally, this combination typically leads to challenges in adhering to necessary treatments, further amplifying the risk of unfavorable clinical outcomes (2, 3). Patients with CI receiving MHD typically experience subtle symptoms that may be overlooked by both their family members and physicians. Therefore, it is crucial to shed light on the influential factors contributing to CI in such patients, aiming to remarkably promote the early prediction and intervention of CI.

Protein-energy wasting (PEW) refers to a condition characterized by progressive malnutrition, which is marked by a decline in plasma albumin levels, excessive consumption of muscle proteins, a state of microinflammation, and in severe cases, cachexia (4). Previous research has indicated a strong association between PEW and children’s cognitive development (5). Additionally, it has been found to be closely correlated with CI in the elderly. While these findings suggest a potential link between PEW and CI, the mechanisms remain unclear, particularly in MHD patients. The conflicting evidence exists regarding whether PEW exacerbates CI through systemic inflammation mediated by pro-inflammatory cytokines (e.g., IL-6, TNF-α) or if CI itself contributes to malnutrition via reduced dietary intake. Chronic inflammation in PEW may disrupt synaptic plasticity through oxidative stress, while uremic toxin accumulation (e.g., p-cresol, indole) could impair neurotransmitter synthesis (6). This study aims to clarify these controversies by leveraging a comprehensive assessment of both nutritional and cognitive parameters in a well-defined MHD cohort. By integrating biomarkers of inflammation (e.g., CRP), metabolic disturbances (e.g., urea clearance), and cognitive function scores (MoCA-B), this work provides mechanistic insights into how PEW-driven pathophysiology contributes to CI progression.

Numerous indications point to the significance of PEW in the progression of CI. However, the association between PEW and CI in patients undergoing MHD remains largely unexplored. Although PEW has been extensively studied in children and elderly populations, its association with cognitive impairment (CI) in patients undergoing maintenance hemodialysis (MHD) remains underexplored. Most prior studies have focused on PEW’s impact on nutritional status, but the direct relationship between PEW and CI, along with its underlying mechanisms—such as chronic inflammation, uremic toxin accumulation, and metabolic disturbances—remains poorly understood, particularly in the MHD population. To address this gap, this study is the first to systematically evaluate the association between PEW and CI in MHD patients by integrating nutritional indicators with cognitive function scores. By elucidating how PEW contributes to CI progression, this work offers novel insights for early prediction and intervention strategies.

## 2 Subjects and methods

### 2.1 Subjects

A total of 185 patients who underwent MHD at the Hemodialysis Center, Department of Nephrology of Army Medical Center of PLA between June 2020 and April 2022 were recruited. Patients were consecutively recruited over a 20-month period (June 2020–April 2022) to minimize selection bias. Inclusion criteria ensured a representative sample of MHD patients, while exclusion criteria (e.g., severe cachexia, visual/hearing impairments) were applied to maintain data quality. The inclusion criteria were as follows: (1) patients who aged 18 years or older; (2) receiving MHD at our center regularly for a minimum of 3 months, 2 times per week, and for a duration of 4 h each session; (3) having a clear and conscious mind, without any communication difficulties; and (4) providing signed informed consent and expressing willingness to participate in the present study. The exclusion criteria were as follows: (1) patients with mental disorders; (2) patients with either history or presence of co-morbidities; (3) patients with hematological diseases; (4) patients with severe cachexia; (5) taking lipid-lowering drugs; (6) patients who were unable to cooperate with the study due to visual and hearing impairments, limb deficiencies, and reduced mobility. Among the initially recruited patients, 25 patients were excluded, and 160 patients were finally involved in this study. The present study was approved by the Ethics Committee of the Army Medical Center of PLA (Approval No. (2022) No. 222). All subjects signed the informed consent form prior to enrollment.

### 2.2 Methods

#### 2.2.1 Baseline data

##### 2.2.1.1 Questionnaire design

The questionnaire was designed by members of our research team with involvement of patients’ gender, age, educational level, and hemodialysis duration.

#### 2.2.2 Laboratory indicators

Fasting venous samples were collected before the hemodialysis to determine the following indicators: albumin (ALB), prealbumin (PAB), cholesterol (CHOL), and urea clearance index (Kt/V).

#### 2.2.3 Measurement of the human body

Height, weight, arm muscle circumference (AMC), triceps skinfold (TSF) thickness, and grip strength were measured by the same researcher at the end of the hemodialysis on the contralateral side of the vascular access. AMC measurement: The distance from the acromion of the scapula to the olecranon was measured. The mid-point of this segment was treated as the mid-point of the upper arm, and the circumference at the mid-point of the upper arm was measured. Measurement of the TSF thickness: The thickness of a pinch of skin on the triceps was measured using an adipometer. Measurement of the grip strength: Patients were instructed to hold the pinch gage in an upright position, while the value was recorded when their index finger’s metacarpophalangeal joint was bent at a right angle. Other indicators were calculated as follows: body mass index (BMI) = weight/height^2^ (kg/m^2^); AMC (cm) = [arm circumference (AC)](cm)-3.14 × TSF (cm); lean body mass = (1 = male;0 = female) × 3.81 + height (cm) × 0.34 + weight (kg) × 0.18 + AMC (cm) × 0.67 + grip strength (N) × 0.02-hemodialysis duration (month) × 0.05–43.47. The aforementioned indicators were repeatedly measured three times, and their average values were subsequently recorded.

#### 2.2.4 Dietary assessment

Over a period of three consecutive days, professionals at the nutrition department of our hospital conducted dietary assessment in accordance with the dietary reference intake (7). Normalized dietary protein intake (nDPI) and normalized dietary energy intake (nDEI) were calculated.

#### 2.2.5 Diagnosis and assessment of PEW

##### 2.2.5.1 PEW diagnosis

According to the 2008 International Society of Renal Nutrition and Metabolism (ISRNM) diagnostic criteria for PEW (8), the diagnosis of PEW was made if the following three criteria were met (at least one characteristic in each category was met): (1) Biochemical indicators: serum ALB level less than 38 g/L; pre-albumin (PAB) level less than 300 mg/L; serum total CHOL level less than 2.59 mmol/L; (2) Weight: BMI less than 23 kg/m^2^; weight loss greater than 5% within 3 months or greater than 10% within 6 months; body fat percentage less than 10%; (3) Muscle mass: reduction in muscle mass greater than 5% within 3 months or greater than 10% within 6 months; reduction in AMC greater than 10% of the median AMC in the same population.

##### 2.2.5.2 PEW assessment

The nutritional status was assessed using the Scored Patient-Generated Subjective Global Assessment (PG-SGA) (9) and Malnutrition Inflammation Score (MIS) (10). The PG-SGA comprises seven distinct domains: weight loss, food intake, nutrition impact symptoms, activities and function, disease, metabolic demand, and physical examination. Each domain is assessed on a scale of 1–5 points, with higher scores representing more severe issues in each domain. Consequently, the total PG-SGA score ranges from 7 to 35 points, in which a higher score indicates poorer nutritional status. The MIS consists of scores for 10 items, including clinical history, body mass index, physical examination, and laboratory indicators. Each item is scored from 1 to 3 points, yielding a total MIS score ranging from 0 to 30 points. A higher MIS score indicates a worse nutritional status. The 2008 ISRNM criteria for PEW have been validated in MHD populations, ensuring consistency in diagnosis. While no specific validation studies were conducted in Chinese MHD patients, the criteria are widely accepted globally, and our sample met the international standards, enhancing the generalizability of the results.

#### 2.2.6 Assessment of cognitive function

The cognitive functions of patients were evaluated using the Montreal Cognitive Assessment-Basic (MoCA-B), which was designed to adapt to populations with different educational levels (11). The MoCA-B has been validated in ESRD patients. The rationale for using this scoring system to identify CI is due to its ability to adapt to different educational levels and assess various cognitive domains, such as executive function, immediate recall, fluency, orientation, calculation, abstraction, delayed recall, visuoperception, naming, and attention. The test duration of 15 min and maximum total score of 30 points make it efficient and comprehensive. Additionally, the MoCA-B considers literacy by adding 1 point to the score of illiterate subjects, regardless of their educational levels. A score of 26–30 indicates normal cognitive function, whereas a score of 0–26 represents CI. The lower the total score, the more severe the CI. The MoCA-B cutoff of 26 was derived from studies in ESRD populations. However, its applicability in Chinese MHD patients with lower educational levels requires caution. To address this, we conducted subgroup analyses stratified by education level to validate the threshold’s relevance.

### 2.3 Statistical analysis

Data were analyzed using the SPSS 23.0 software (IBM, Armonk, NY, United States). Qualitative data were described as mean and percentage (%), and quantitative data were presented as mean ± standard deviation ( $\bar{x}$ ± s). Qualitative data were compared between the CI and non-CI groups using the *χ*^2^ test. Quantitative data were compared using the independent-samples *t*-test. The analysis concentrated on univariate Spearman correlations. Factors that had a significance level of *p* < 0.05 were included in the subsequent logistic regression analysis. This analysis aimed to identify independent factors that could influence the cognitive functions of patients undergoing MHD. A significance level of α = 0.05 was set, and *p*-value below 0.05 was considered statistically significant. The sample size was calculated based on the prevalence of CI (42.5%) and PEW (32.5%) from a pilot study, using the formula for a cross-sectional study. A sample size of 160 was determined to achieve 80% statistical power at α = 0.05. Despite the relatively small sample size, strict inclusion/exclusion criteria and multivariable analysis were applied to control confounding factors, ensuring the validity of the findings. Additionally, subgroup analyses were performed based on sex (male vs. female) and dialysis duration (<3 years vs. ≥3 years) to explore potential differences in CI patterns across patient subgroups. Multicollinearity was tested using variance inflation factor (VIF) values, with all VIF scores below 10, indicating no significant multicollinearity.

## 3 Results

### 3.1 Comparison of baseline data and PEW-related indicators in MHD patients

A total of 160 patients were assessed using MoCA-B, and they were divided into CI group (*n* = 68, 42.5%) and non-CI group (*n* = 92, 57.5%). According to the 2008 ISRNM diagnostic criteria for PEW, the detection rate of PEW was estimated. This refers to the proportion of MHD patients with PEW among all MHD patients. It was found that the PEW detection rate was significantly higher in the CI group than that in the non-CI group [60.29% (41/68) vs. 11.96% (11/92)] (*p* < 0.05). Significant differences were observed between the CI and non-CI groups in baseline characteristics and PEW-related indicators, including age, educational level, Kt/V, PEW detection rate, and key PEW indicators (albumin [ALB], lean body mass, arm muscle circumference [AMC], normalized daily protein intake [nDPI], Scored Patient-Generated Subjective Global Assessment [PG-SGA] score, and Malnutrition-Inflammation Score [MIS] score) (*p* < 0.05) (Table 1).

**Table 1:** Comparison of baseline data and PEW-related indicators in MHD patients (*n* = 160) [*n* (%), $\bar{x}$ ± s].

Items	Non-CI group (*n* = 92)	CI group (*n* = 68)	*t*/χ^2^	*P*
Age (year)	55.03 ± 17.07	64.89 ± 16.00	−2.697	0.019
Male	49 (53.26)	35 (51.47)	0.747	0.504
Hemodialysis duration (month)	50.03 ± 39.78	52.07 ± 39.12	0.634	0.612
Education level (year)	8.29 ± 3.56	4.39 ± 3.41	3.107	0.009
Kt/V (unitless)	1.38 ± 0.29	1.21 ± 0.21	2.992	0.014
PEW detection rate [case (%)]			−4.292	0.002
Not combined with PEW	81 (−)	27 (−)		
Combined with PEW	11 (11.96)	41 (60.29)		
Single characteristic of PEW				
Albumin (g/L)	41.41 ± 4.80	33.89 ± 4.05	2.721	0.017
Prealbumin (mg/L)	309.57 ± 72.90	287.51 ± 65.11	0.782	0.541
Total cholesterol (mmol/L)	4.53 ± 0.78	4.42 ± 0.72	0.602	0.632
BMI (kg/m^2^)	23.78 ± 3.45	21.28 ± 3.09	1.321	0.098
Lean body mass (kg)	48.09 ± 6.80	40.78 ± 5.89	2.023	0.041
Arm muscle circumference (cm)	24.53 ± 3.09	19.01 ± 2.67	2.387	0.036
nDPI[g/(kg·d)]	0.95 ± 0.31	0.70 ± 0.28	2.626	0.029
nDEI[kcal/(kg·d)]	32.27 ± 7.89	28.08 ± 6.33	0.883	0.351
PG-SGA score	9.87 ± 3.73	17.45 ± 5.67	−2.992	0.010
MIS score	4.31 ± 2.26	14.67 ± 4.77	−3.853;	0.005

CI, cognitive impairment; Kt/V, urea clearance index; PEW, protein energy wasting; BMI, body mass index; ALB, Albumin; nDPI, normalized daily protein intake; nDEI, normalized daily energy intake; PG-SGA, Scored Patient-Generated Subjective Global Assessment; MIS, Malnutrition-Inflammation Score.

### 3.2 Subgroup analysis by sex and dialysis duration

Subgroup analyses revealed significant differences in CI prevalence and PEW severity between male and female patients (Table 2). Male patients had a higher PEW detection rate (65.2% vs. 52.3%, *p* = 0.021) and lower MoCA-B scores (18.4 ± 4.1 vs. 22.1 ± 4.7, *p* = 0.008) compared to female patients. Among patients with different dialysis durations, those with ≥3 years of dialysis showed a higher PEW detection rate (68.9% vs. 45.6%, *p* = 0.013) and more severe cognitive impairment (MoCA-B score: 17.3 ± 4.3 vs. 24.5 ± 4.6, *p* < 0.001) (Table 2). Additionally, subgroup analysis by education level showed that patients with <6 years of education had a higher PEW detection rate (66.7% vs. 44.2%, *p* = 0.001) and significantly lower MoCA-B scores (18.2 ± 4.3 vs. 24.5 ± 4.6, *p* = 0.001), highlighting the need for a region-specific cutoff for this subgroup.

**Table 2:** Subgroup analysis of CI and PEW by sex and dialysis duration (*n* = 160).

Subgroup	CI group (*n* = 68)	Non-CI group (*n* = 92)	PEW detection rate (%)	MoCA-B score (points) (x̄±s)	*P*-value
Sex					
Male	41 (60.3%)	35 (38.0%)	65.2	18.4 ± 4.1	0.021
Female	27 (39.7%)	57 (62.0%)	52.3	22.1 ± 4.7	
Education level					0.001
<6 years	12 (17.6%)	15 (16.3%)	66.7	18.2 ± 4.3	
≥6 years	56 (82.4%)	77 (83.7%)	44.2	24.5 ± 4.6	
Dialysis duration					
<3 years	18 (26.5%)	49 (53.3%)	45.6	24.5 ± 4.6	0.013
≥3 years	50 (73.5%)	43 (46.7%)	68.9	17.3 ± 4.3	

CI, cognitive impairment; MoCA-B, montreal cognitive assessment-basic; PEW, protein energy wasting.

### 3.3 Comparison of the cognitive function scores in patients undergoing MHD with and without PEW

**Figure 1:**
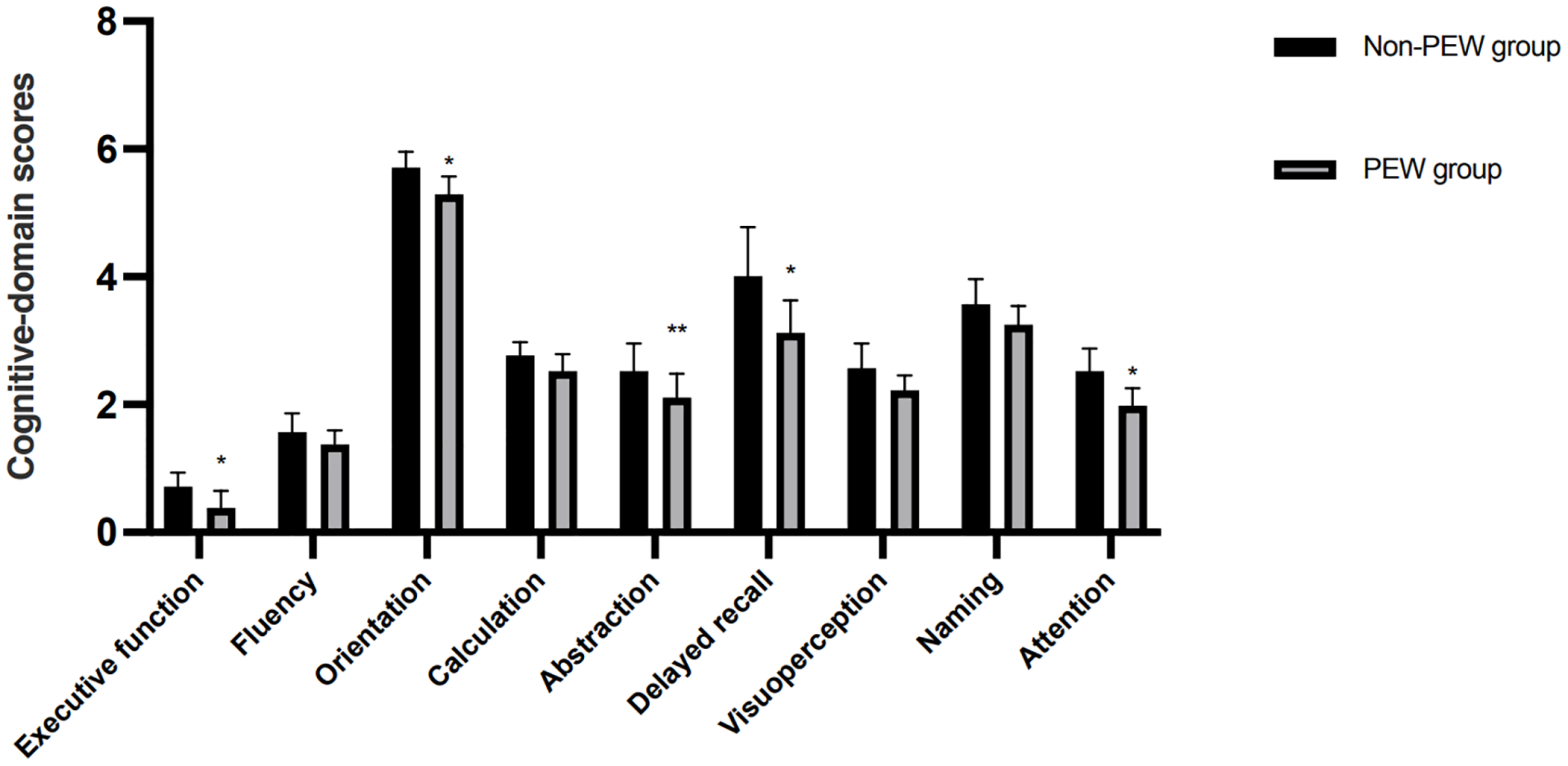
Cognitive-domain scores in MHD patients with and without PEW. *p < 0.05, PEW vs. non-PEW groups; **p < 0.01, PEW vs. non-PEW groups.

The total MoCA-B score was significantly lower in patients with PEW (19.05 ± 4.27) compared to those without PEW (25.79 ± 4.55) (*p* < 0.05). The effect size (Cohen’s d = 1.37) indicated a large clinical impact of PEW on cognitive function (Table 3). The 6.74-point decrease in MoCA-B scores among PEW patients may impair daily functioning, such as medication adherence. A bar chart (Figure 1) illustrating the differences in cognitive-domain scores between PEW and non-PEW groups is provided, with executive function (0.38 vs. 0.72, *p* = 0.002) and attention (1.98 vs. 2.52, *p* = 0.003) showing the most pronounced declines.

**Table 3:** Comparison of the cognitive functions in MHD patients with and without PEW ( $\bar{x}$ ± s).

Items	Not combined with PEW (*n* = 108)	Combined with PEW (*n* = 52)	*t*/χ^2^	*P*
Executive function	0.72 ± 0.22	0.38 ± 0.27	7.289	0.002
Fluency	1.57 ± 0.29	1.38 ± 0.22	2.237	0.139
Orientation	5.71 ± 0.25	5.29 ± 0.28	3.081	0.019
Calculation	2.77 ± 0.21	2.52 ± 0.27	1.490	0.378
Abstraction	2.52 ± 0.44	2.11 ± 0.37	3.872	0.014
Delayed recall	4.01 ± 0.77	3.12 ± 0.51	4.225	0.007
Visuoperception	2.57 ± 0.34	2.22 ± 0.24	2.012	0.153
Naming	3.57 ± 0.39	3.25 ± 0.29	1.815	0.269
Attention	2.52 ± 0.36	1.98 ± 0.28	6.102	0.003
Total score	25.79 ± 4.55	19.05 ± 4.27	5.513	0.004

PEW, protein-energy wasting; MoCA-B, montreal cognitive assessment-basic.

### 3.4 Univariate correlation analysis of factors associated with CI in MHD patients

Univariate Spearman correlation analysis revealed significant associations between CI and several variables. Age, PEW detection rate, PG-SGA score, and MIS score were positively correlated with CI (*r* = 0.232, 0.322, 0.201, and 0.269, respectively; *p* < 0.05). Conversely, educational level, Kt/V, ALB level, and lean body mass were negatively correlated with CI (*r* = −0.173, −0.192, −0.212, and −0.177, respectively; *p* < 0.05) (Table 4).

**Table 4:** Univariate correlation analysis between CI and PEW-related indicators in MHD patients (r values and *p* values).

Items	Statistics	
	Spearman’s r	*P*-value
Age	0.232	0.007
Education level	−0.173	0.021
Diabetic nephropathy	0.091	0.207
Kt/V	−0.192	0.019
PEW detection rate	0.322	0.004
Single characteristic of PEW		
Albumin	−0.212	0.011
Lean body mass	−0.177	0.019
Arm muscle circumference (AMC)	−0.087	0.256
nDPI[g/(kg·d)]	−0.062	0.391
PG-SGA score	0.201	0.013
MIS score	0.269	0.006

Kt/V, urea clearance index; PEW, protein energy wasting; nDPI, normalized daily protein intake; PG-SGA, Scored Patient-Generated Subjective Global Assessment; MIS, Malnutrition-Inflammation Score; CI, cognitive impairment; ALB, Albumin.

### 3.5 Multivariate logistic regression analysis of factors associated with CI in MHD patients

Multivariate logistic regression analysis identified independent predictors of CI in MHD patients. Age, PEW severity (measured by PEW detection rate, PG-SGA score, and MIS score), Kt/V, and ALB level were significantly associated with CI (OR = 4.860–5.842, *p* < 0.05) (Table 5).

**Table 5:** Multivariate logistic regression analysis of factors associated with CI in MHD patients (OR values and 95% CI).

Items	Statistics			
	β-value	*OR*	*P*-value	95% CI
Age	1.581	4.860	0.014	1.377–17.151
Education level	–	–	0.021	
Kt/V	−1.421	0.241	0.035	0.064–0.905
PEW detection rate	1.765	5.842	0.016	1.389–24.559
Single characteristic of PEW				
Albumin	−1.201	0.301	0.039	0.096–0.941
Lean body mass	–	–	0.027	
PG-SGA score	1.342	3.827	0.019	1.247–11.745
MIS score	1.251	3.494	0.036	1.085–11.249

Kt/V, urea clearance index; PEW, protein energy wasting; PG-SGA, Scored Patient-Generated Subjective Global Assessment; MIS, Malnutrition-Inflammation Score; CI, cognitive impairment; ALB, Albumin.

## 4 Discussion

CI is one of the most common co-morbidities of MHD. A study demonstrated that the incidence of CI was 24–67% in patients undergoing MHD (12), which is in agreement with the incidence achieved in the present study (42.5%). One prospective study on CI and the 7-year survival among patients undergoing MHD showed that CI was an independent risk factor for mortality (13). Notably, CI in patients undergoing MHD is typically accompanied by occult symptoms that are mainly overlooked by both relatives and physicians. Therefore, it is crucial to identify the influential factors of CI in such patients, as it can remarkably promote the early prediction and intervention of CI.

PEW is a condition characterized by a decrease in the body’s storage of protein and energy fuels. This can lead to various clinical manifestations, such as abnormal laboratory indicators, low BMI, reduced muscle content, a state of microinflammation, and insufficient intake of dietary nutrients and energy (4). Research has indicated that the incidence of PEW in patients undergoing MHD ranges from 22 to 75%. It has been identified as a significant factor that negatively impacts the quality of life and is associated with all-cause mortality (14, 15). To date, there have been limited studies on the relationship between PEW and CI in patients undergoing MHD. In the present study, an initial screening of PEW-positive patients undergoing MHD was conducted using the 2008 ISRNM diagnostic criteria for PEW. The detection rate of PEW was found to be 32.5%, aligning with previously reported findings (7). Subgroup analysis revealed that the PEW detection rate was significantly higher in the CI group (60.29%) compared with the non-CI group. To further evaluate nutritional status, the assessment using PG-SGA (9) and MIS (10) scores was conducted. The CI group exhibited significantly higher PG-SGA and MIS scores compared with the non-CI group. Moreover, there was a significantly positive correlation between the PEW detection rate, PG-SGA score, MIS score, and CI. These findings strongly indicated a close association between PEW and CI in patients undergoing MHD, highlighting the elevated vulnerability of malnourished individuals to CI. In a previous study (16), PEW was found as a possible independent risk factor for consciousness dysfunction in patients undergoing MHD. The present study yielded similar findings, demonstrating that the PG-SGA and MIS scores in the CI group were significantly higher than those in the non-CI group. The subgroup analyses highlighted significant variations in CI patterns between male and female patients, as well as across different dialysis durations. Male patients exhibited a higher PEW detection rate and more severe cognitive impairment, which may be attributed to gender-specific differences in muscle mass and hormonal factors. Patients with longer dialysis durations showed greater PEW severity, likely due to cumulative nutritional losses and chronic inflammation. These findings underscore the importance of tailored interventions for high-risk subgroups, such as prolonged dialysis patients and males, to mitigate CI progression.

While this study primarily focused on the impact of PEW on CI, the possibility of a reverse association—where CI exacerbates PEW—deserves critical analysis. For instance, CI may impair patients’ ability to adhere to dietary recommendations or recognize symptoms of malnutrition, creating a vicious cycle. A study in elderly patients with Alzheimer’s disease found that cognitive decline was associated with reduced food intake and weight loss, suggesting a bidirectional relationship between cognitive function and nutritional status (17). Similarly, in MHD patients, CI could lead to poor medication adherence, reduced appetite, or difficulty in self-care, all of which may accelerate the progression of PEW. This reverse association is further supported by the observation that patients with CI in our study had lower lean body mass and albumin levels, which are key indicators of PEW. However, the cross-sectional design of this study limits our ability to determine the directionality of this relationship. Future longitudinal studies are needed to clarify whether CI precedes PEW or vice versa, as well as to explore the underlying mechanisms, such as neuroendocrine changes or behavioral factors. Socio-economic status, a potential unmeasured confounder, may influence both nutritional intake and cognitive function in MHD patients. Future studies should incorporate socioeconomic indicators to further clarify these associations.

Comparative studies in Western populations (e.g., European or North American cohorts) have reported similar PEW prevalence (22–75%) (18), supporting the global relevance of our findings. However, cultural differences in dietary habits and healthcare access may influence the severity of PEW and CI in Chinese MHD patients, warranting further investigation. For example, while Western studies often emphasize protein intake and caloric balance as key drivers of PEW, our results highlight the role of microinflammation and uremic toxin accumulation in the Chinese context. Additionally, the MoCA-B cutoff of 26, derived from ESRD populations, may require adjustment for Chinese MHD patients with lower educational levels, as evidenced by our subgroup analyses. These differences underscore the importance of region-specific approaches to PEW and CI management in MHD patients.

In accordance with the 2008 ISRNM diagnostic criteria for PEW, the present study incorporated various quantitative indicators, including laboratory markers (e.g., ALB, PAB, and CHOL), lean body mass, AMC, nDPI, and nDEI. The findings revealed significant differences between the CI and non-CI groups. Specifically, the CI group exhibited significantly lower levels of ALB, lean body mass, AMC, and nDPI compared with the non-CI group. Furthermore, ALB and lean body mass displayed a significantly negative correlation with CI. Besides, the multivariate logistic regression analysis demonstrated ALB as an influential factor of CI progression in patients undergoing MHD. ALB, a protein carrier and an antioxidant in organisms, may play an important role in CI by acting on neurotransmitter transmission and inflammation activation (19, 20). Lean body mass, which refers to the weight of the human body excluding fat mass, encompasses all non-fat components. Among these components, muscles constitute the largest proportion of lean body mass. A higher lean body mass indicates a greater muscle development and overall muscularity. In a study conducted by Noh et al. (21), body compositions of 320 elderly patients were analyzed, and it was found that lean body mass played a significant role in impairing cognitive functions, which are consistent with our findings. One possible explanation is that prolonged low lean body mass exacerbates muscular atrophy and functional decline, ultimately leading to cerebral atrophy caused by the progressive reduction of limb mobility. Efforts can be undertaken to enhance the dietary structure of patients undergoing MHD and increase their consumption of high-quality proteins. Additionally, patients may receive intravenous infusion of ALB to elevate their serum ALB level. Furthermore, providing guidance on physical exercises to patients undergoing MHD can enhance their muscle mass and slow down the progression of CI. Another notable characteristic of PEW is the chronic microinflammatory state induced by the prolonged depletion of nutrients. The MIS scores indicated that the PEW-related chronic microinflammatory state was closely associated with CI in patients undergoing MHD. Increased levels of proinflammatory factors, such as interleukin-1β (IL-1β), tumor necrosis factor-α (TNF-α), and IL-6 may lead to continual oxidative stress, resulting in injury to microvascular endothelial cells and the death of neuronal cells (22).

Besides, we conducted an analysis to examine the relationship between baseline data and CI in patients undergoing MHD. The results revealed significant correlations between CI and age, educational level, and Kt/V in patients undergoing MHD. Furthermore, multivariate logistic regression analysis demonstrated that age and Kt/V were influential factors of CI in patients undergoing MHD. These findings are consistent with research conducted by Chen et al. (23), which also demonstrated that advanced age is a risk factor for CI in patients undergoing MHD. A recent study indicated that the deterioration of cerebral cortical and subcortical network connections due to aging plays a significant role in the progression of CI (24). These findings align with our findings, as Hou et al. also reported a strong association between educational level and CI in patients undergoing MHD. This relationship may be attributed to the influence of education on language skills, cognitive processes, orientation, and memory abilities. The higher the educational level, the greater the brain activities, demonstrating a wider threshold for the incidence of CI (25). Kt/V has been widely recognized as a measure for dialysis adequacy (26). Its influence on the progression of CI may be explained by the fact that increased dialysis adequacy leads to the mitigation of vascular calcification (27) and inhibition of inflammatory activation (28). The calcification of peripheral vessels can potentially worsen renal function impairment, leading to elevated levels of blood toxins, such as creatinine. This increased creatinine level, along with the presence of inflammatory factors, may together cause damage to cortical neurons.

According to the above-mentioned results, PEW was closely associated with CI in patients undergoing MHD. While this study identifies a strong association between PEW and CI, the cross-sectional design limits causal inference. Future longitudinal studies are needed to determine whether PEW precedes CI or vice versa. Notably, it is possible that patients with CI would be more prone to the development of PEW. Therefore, active precautionary measures should be taken against PEW to delay the progression of CI in patients undergoing MHD. Clinical interventions should focus on optimizing nutritional status through individualized dietary plans, intravenous albumin supplementation, and structured exercise programs. Additionally, regular cognitive monitoring using tools like MoCA-B is essential for early detection of CI in high-risk patients. Age and Kt/V were identified as risk factors of CI that contributed to the progression of CI in such patients. One limitation of this study was that the present study was a single-center cross-sectional study, with a limited sample size. In addition, the current study only reported the above-mentioned association, while it did not present any direction of association. In the future research, it is crucial to enroll a greater number of patients for further validation, enhancing the reliability of our findings.

## Data Availability

The original contributions presented in the study are included in the article/supplementary material, further inquiries can be directed to the corresponding author.
